# Effects of the Surface Charge of Graphene Oxide Derivatives on Ocular Compatibility

**DOI:** 10.3390/nano12050735

**Published:** 2022-02-22

**Authors:** Liyuan Rong, Yan Fu, Qiyou Li, Xinji Yang, Yueyue Li, Liang Yan, Liqiang Wang, Wei Wu

**Affiliations:** 1 Senior Department of Ophthalmology, The Third Medical Center of PLA General Hospital, Beijing 100143, China; liyuan_rong@outlook.com (L.R.); yangxinji68@sina.com (X.Y.); liyueyue_7909@163.com (Y.L.); 2 Department of Ophthalmology, Southwest Hospital, Third Military Medical University (Army Medical University), Chongqing 400038, China; lqy111lcy@126.com; 3 The General Hospital of Western Theater Command, Chengdu 610000, China; fuyansmile@163.com; 4 CAS Key Laboratory for Biomedical Effects of Nanomaterials and Nanosafety, Institute of High Energy Physics and National Center for Nanoscience and Technology, Chinese Academy of Sciences, Beijing 100049, China

**Keywords:** surface charge, graphene oxide, nanotoxicology, biocompatibility, nano-bio interactions

## Abstract

The incorporation of functional groups endows graphene oxide (GO) with different surface charges, which plays important roles in biological interactions with cells. However, the effect of surface charge of GO derivatives on ocular biocompatibility has not been fully elucidated. Previously, we found that positively, negatively and neutrally charged PEGylated GO (PEG-GO) nanosheets exerted similar effect on the viability of ocular cells. In this work, we performed in vitro and in vivo studies to comprehensively study the effect of surface charge of PEG-GO on ocular compatibility. The in vitro results showed that the cellular uptake efficacy of negatively charged PEG-GO nanosheets was significantly decreased compared with positively charged and neutrally charged analogs. However, three kinds of PEG-GO nanosheets produced similar amounts of intracellular reactive oxygen species and showed similar influence on mitochondrial membrane potential. By analysis of global gene expression profiles, we found that the correlation coefficients between three kinds of PEG-GO-treated cells were more than 0.98. Furthermore, in vivo results showed that all these PEG-GO nanosheets had no significant toxicity to ocular structure and function. Taken together, our work suggested that surface charge of PEG-GO exerted negligible effect on its ocular compatibility, except for the cellular uptake. Our work is conducive to understanding the relationship between surface charge and biocompatibility of GO derivatives.

## 1. Introduction

Graphene-based nanomaterials have promoted advancements in biomedical fields due to their unique nanoscopic properties and tunable multiple functions [1,2,3]. In particular, graphene oxide (GO) and its derivatives exhibit infinite potential for drug delivery applications [2,3,4,5,6]. In order to improve the efficiency of drug delivery, as a drug platform, GO is usually incorporated by different reactive groups [1,3,6]. The incorporation of functional groups endows GO with different surface charges, which can influence the cellular uptake and drug delivery efficiency [7,8,9,10]. However, the effect of surface charge of GO derivatives on nanotoxicology remains controversial so far.

Tu et al. found that the positively charged reduced GO nanosheets possessed higher cellular uptake efficiency, as well as higher cytotoxicity, than negatively and neutrally charged counterparts using human breast cancer cell line (MCF7 cells) as the experimental model [7]. However, Wang et al. suggested that positively charged GO derivatives showed higher intracellular delivery efficiency but similar nanotoxicity compared with negatively charged counterparts using mouse macrophage-like cell line (Raw 264.7 cells) as the experimental model [11]. Studies on gold nanorods consistently showed that positive charge tended to enhance cellular uptake, but surface charge alone displayed negligible effect on the cytotoxicity [12,13]. On the other hand, Bhattacharjee et al. suggested that positively charged Si core nanoparticles were more cytotoxic in terms of reducing mitochondrial metabolic activity and producing intracellular reactive oxygen species (ROS) [14]. The possible causes of these controversies could contribute to the intrinsic differences among the different nanomaterials. More importantly, the confounding variables other than surface charge, such as size, shape, surface chemistry and aggregation, could also make a difference [7,15,16]. As a result, when studying the relationship between specific properties of nanomaterials and their cytotoxicity, it is of the utmost importance to minimize the effect of these confounding variables and ensure a fair comparison.

In recent years, extensive studies have shown that GO-based nanomaterials hold great potential application in the ocular surface and intraocular diseases [17,18,19,20,21]. As a result, it is increasingly important to comprehensively evaluate the ocular toxicity of GO derivatives. Previously, we studied the relationships between physico-chemical properties of PEGylated GO (PEG-GO) and the resulting ocular toxicity and focused on the oxidation-state-induced toxicity of PEG-GO [22]. Although we have demonstrated that PEG-GO nanosheets with different surface charges exert similar toxicity in terms of cell viability [22], the effect of surface charge of PEG-GO on ocular toxicity remains unclear. In this study, we further comprehensively analyzed the cellular response of PEG-GO with different surface charges in terms of cellular uptake, ROS generation, mitochondrial membrane potential (MMP) and global gene expression profiles on human corneal epithelial cells (hCorECs) and human retinal capillary endothelial cells (hRCECs) in vitro. In addition, we evaluated the nanotoxicology of these GO derivatives on ocular surface and inner eyes of rats in vivo. Our research helps to thoroughly understand the effect of surface charge on the biocompatibility of GO derivatives.

## 2. Materials and Methods

### 2.1. Ethics Statement

All experiments involving human cells and tissues were performed according to the tenets of the Declaration of Helsinki and with informed consent from donors. All animals were housed according to the Third Military Medical University (Army Medical University, Chongqing, China) guidelines. All procedures were approved by the ethics committee of Southwest Hospital and PLA General Hospital.

### 2.2. Cell Culture

According to previous protocol [23], hCorECs were isolated from aborted fetuses (gestation, 13 weeks) with ethics committee approval and donors’ informed consent. Corneal tissue was cut into explants of 5 mm × 5 mm and then placed on fibronectin-coated 6-well plates with epithelium side down. Fetal bovine serum (FBS, 40 μL) was added to cover the explants overnight after 10 min attachment. The cells were then cultured in Dulbecco’s modified Eagle medium (DMEM)/Nutrient Mixture F-12 supplemented with penicillin/streptomycin (5000 units/mL), insulin (1 μg/mL), hydrocortisone (0.5 μg/mL, Sigma-Aldrich, St. Louis, MO, USA), cholera toxin (0.1 μg/mL, Gentaur, Kampenhout, Belgium), EGF (2 ng/mL) and 10% FBS (all from Invitrogen, Carlsbad, CA, USA, unless indicated otherwise) the next day. The hRCECs were purchased from the BeNa Culture Collection (BNCC339792, Beijing, China). hRCECs were cultured in DMEM supplemented with 10% FBS. Both cells were passaged at 1:3 when they reached sub-confluency. hCorECs at passage 1 (P1)–P4 and hRCECs at P3–P6 were used. Then, the hCorECs and hRCECs were plated in 6-well plates and treated with or without negatively charged GO-PEG-NH_2_, neutrally charged GO-PEG-OCH_3_ and positively charged GO-PEG-COOH (50 μg/mL) for 24 h. Cells were collected or assays conducted after 24 h treatment.

### 2.3. Transmission Electron Microscopy (TEM)

The TEM assay was conducted as previously described [23]. The harvested samples (cells or retinas) were successively fixed in 2.5% glutaraldehyde and 1% osmium tetroxide. After fixation, the samples were dehydrated with increasing concentrations of acetone, embedded in propylene oxide and implanted in epoxy resin 618. The samples were then cut into thin sections with an ultra-microtome (Leica, Leica Biosystems, Shanghai, China) and stained with uranyl acetate and lead citrate. After staining, the sections were examined with JEM-1400 Plus (JEOL, Tokyo, Japan) electron microscope at an operating voltage of 120 kV.

### 2.4. Side Scatter (SSC) Analysis

The hCorECs and hRCECs exposed with or without three kinds of PEG-GO nanosheets (50 μg/mL) for 24 h were collected and analyzed using a Fluorescence Activating Cell Sorter (FACS) Calibur Flow Cytometer (BD Bioscience, San Jose, CA, USA). At least 10,000 cells were collected for each sample and analyzed using FlowJo software (Ashland, OR, USA). Three independent experiments were conducted. The SSC value of each sample was then analyzed via FlowJo software (Ashland, OR, USA) with at least 10,000 cells in gate. Mean SSC values reflecting the inner cell granularity and complexity were obtained and averaged as previously described [24,25].

### 2.5. Intracellular ROS and MMP Detection

Serum-starved hCorECs and hRCECs were exposed to three kinds of PEG-GO nanosheets (50 μg/mL) for 24 h, while untreated cells were used as a control. The MMP and ROS detection were performed as described previously [22,26]. The cells were washed with PBS three times and then incubated with 1 μM Rhodamin 123 (C2007, Beyotime, Shanghai, China) and 10 mM H2DCF-DA (20, 70-dichlorofluorescin diacetate) (D6883, Sigma, St. Louis, MO, USA) for 30 min to detect MMP and ROS, respectively. After incubation, cells were washed twice with PBS. The fluorescence intensity was then determined by FACS Calibur Flow Cytometer (BD Bioscience, San Jose, CA, USA) and analyzed using FlowJo software (Ashland, OR, USA). Three independent experiments were conducted.

### 2.6. Gene Expression Profile Analysis with RNA Sequencing (RNA-Seq)

Serum-starved hCorECs treated by three kinds of PEG-GO nanosheets (50 μg/mL) for 24 h were collected to extract the total RNA using Trizol (Invitrogen, Carlsbad, CA, USA). The vehicle (DMEM)-treated hCorECs served as control. Each group had three biological replicates. The library preparations were sequenced on an Illumina Hiseq 2500 platform after library construction. Each sample generated more than 20 M clean reads. The gene analysis was performed as previously described [22]. Briefly, the strict quality control for each sample from several aspects, i.e., clean read Q20 ≥ 95%, clean read Q30 ≥ 90%, clean reads ≥ 20 M, gene unique mapping ratio ≥ 80% and genome mapping ratio ≥ 50%, were performed to evaluate whether the sequencing data were qualified. The Pearson correlation coefficients were based on all gene expression levels. “Fragments per kilobase of transcript per million fragments mapped” (FPKM) was used to compare the differences in gene expression between the treated sample and the control sample. The “NOISeq” method was used to screen differentially expressed genes (DEGs) between two groups, following the default criteria: fold change ≥ 2 and adjusted *p*-value < 0.05. The Venn diagram was imaged with paired comparisons between three PEG-GO-treated groups, as well as comparisons between different PEG-GO-treated groups with control. To obtain the biological pathways of DEGs, an analysis was performed based on the Kyoto Encyclopedia of Genes and Genomes (KEGG) database using Dr. Tom software (http://biosys.bgi.com in 16 December 2021).

### 2.7. Ocular Surface Irritation Test

Sprague Dawley (SD) rats (3-week-old female, *n* = 5 per group) without eye abnormalities were used for ocular surface irritation test. One drop of a topical ocular anesthetic (0.4% oxybuprocaine hydrochloride) was used for each eye 5 min before PEG-GO application to minimize the potential pain and distress. Afterward, 2 μL of PEG-GO samples (50 μg/mL) or deionized water (control) were topically administered to the conjunctival sac of right eye twice a day for two consecutive days. The left eye remained untreated and served as the control. The corneal opacity, conjunctival redness, iris and chemosis of SD rats were evaluated using a slit lamp at 1, 6, 12 and 24 h post PEG-GO treatment. Corneal fluorescein staining assay and scanning electron microscope (SEM) analysis were performed 24 h after PEG-GO treatment. The test was replicated three times.

### 2.8. Corneal Fluorescein Staining

Corneal fluorescein staining was performed according to our previous protocol [23]. Briefly, SD rats were topically administered with 2 μL 3% fluorescein into the conjunctival sac and were then examined with slit lamp using cobalt-blue light 2 min later. The photographs were obtained and analyzed. The test was replicated three times.

### 2.9. SEM Analysis

SEM analysis was performed as previously described [23]. In brief, SD rats were killed with overdosed sodium pentobarbital (Sigma-Aldrich). The enucleated eyeballs were then fixed in 2.5% glutaraldehyde overnight. After washing twice with 0.9% NaCl, eyeballs were dehydrated with graded ethanol and tert-butyl alcohol. Finally, the corneas were critical point-dried, silver plated and analyzed using a JSM-6400 scanning electron microscope (JEOL). The test was replicated three times.

### 2.10. Intraocular Irritation Test

Long Evens (LE) rats (3-week-old-female, *n* = 5 per group) without eye abnormalities were anesthetized with an intraperitoneal injection of 1% pentobarbital sodium (50 mg/kg) and topically anesthetized with a drop of 0.4% oxybuprocaine hydrochloride. Afterward, 2 μL of PEG-GO samples (50 μg/mL) or deionized water (control) were injected into the vitreous body of the right eye through the pars plana with a 33-gauge syringe (Hamilton Storage, Franklin, MA, USA). The left eye remained untreated and served as the control. The responses, such as infection or hemorrhage, were observed using fundus photography at 1, 3 and 7 d post PEG-GO treatment. Electroretinogram (ERG) and TEM analysis were performed 4 weeks after PEG-GO treatment. The test was replicated three times.

### 2.11. ERG Recording

ERG recording was performed to estimate the visual function as previously described [23]. In brief, LE rats were dark adapted overnight for at least 12 h and anesthetized as before. Pupils were dilated with 1% tropicamide. Two gold wire loops were placed on each cornea simultaneously as the recording electrodes, whereas two needle electrodes were placed subcutaneously in the mid-frontal area of the head and tail as the reference and ground electrodes, respectively. The rats were subjected to flashes of −20 dB and 0 dB using a Reti-scan system (Roland consult, Brandenburg, Germany). The amplitudes of a- and b-waves were recorded and analyzed. All the procedures were performed in a dark room with dim red safety light.

### 2.12. Immunofluorescence Staining

The preparations and immunofluorescence staining of retina tissue were performed according to our previous protocol [27]. Briefly, the eyes were prefixed in 4% paraformaldehyde (PFA) at room temperature for 30 min. The anterior segments were then removed and fixed in 4% PFA for an additional 2 h. The retinas were then extracted and flattened onto glass slides. After being permeabilized with 0.3% Triton X-100 (Beyotime, Shanghai, China) in PBS for 15 min and blocked in 3% bovine serum albumin for 60 min, the primary antibodies against Neuronal Class III β-Tubulin (Tuj1, Sigma-Aldrich, St. Louis, MO, USA) were diluted in the same blocking buffer and incubated with the samples overnight at 4 °C followed by incubation with secondary antibodies for 1 h at 37 °C. Nuclei were counterstained with 4′,6-diamidino-2-phenylindole (DAPI; Sigma-Aldrich). Confocal images were obtained using a confocal microscopy system (Zeiss LSM 800, Oberkochen, Germany).

### 2.13. Statistical Analysis

All experiments were individually repeated at least three times. Data were presented as the mean ± standard deviation (S.D.) and plotted with GraphPad Prism 6.0c. Unless mentioned otherwise, the multi-comparisons were performed using analysis of variance (ANOVA) followed by Tukey’s protected least-significant difference post hoc test using SPSS 22.0 (Chicago, IL, USA). *p* < 0.05 was considered statistically significant.

## 3. Results

### 3.1. Cellular Uptake

PEG-GO nanosheets with different surface charges were prepared according to our previous study, that is GO-PEG-NH_2_, GO-PEG-COOH and GO-PEG-OCH_3_ with positive, negative and neutral surface charges, respectively [22]. Atomic force microscopy images showed that the lateral size of PEG-GO samples was about 65 nm, and the thickness was about 2 nm (supplementary materials Figure S1). The physico-chemical properties, including zeta potential at different pH values and hydrodynamic diameter in water and DMEM, were listed in Table 1, respectively [22].

**Figure 1:**
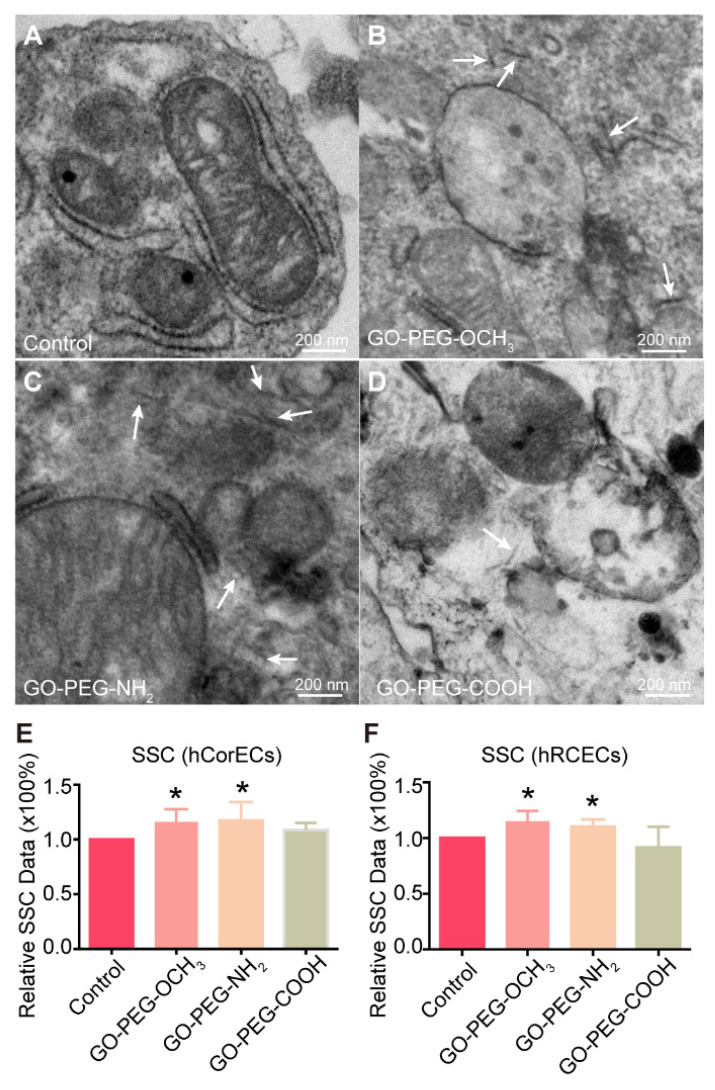
Cellular uptake of PEGylated graphene oxide (PEG-GO) nanosheets with different surface charges by human corneal epithelial cells (hCorECs) and human retinal capillary endothelial cells (hRCECs). (**A**–**D**) The ultrastructure of hCorECs exposed to (**A**) control (0 mg/mL), (**B**) neutrally (GO-PEG-OCH_3_), (**C**) positively (GO-PEG-NH_2_) and (**D**) negatively (GO-PEG-COOH) charged PEG-GO samples (50 μg/mL), respectively. Scale bar = 200 nm. White arrows indicate the internalized PEG-GO nanosheets. (**E**,**F**) Side scatter (SSC) analysis of (**E**) hCorECs and (**F**) hRCECs treated by PEG-GO nanosheets. Compared with control group: * *p* < 0.05. Bars show mean ± standard deviation (S.D.). SSC depends on the inner granularity and complexity of the cells.

Firstly, we used TEM to qualitatively detect the ultrastructure of hCorECs exposure to PEG-GO samples for 24 h. Compared with the untreated cells (Figure 1A), there were some typically needle-shaped objects (indicated by white arrows) with lateral size of about 65 nm detected in the cytoplasm of PEG-GO-treated hCorECs (Figure 1B–D). These data demonstrated that the three kinds of PEG-GO samples with different surface charges could be taken up by cells. Notably, the needle-shaped PEG-GO nanosheets frequently appeared in GO-PEG-OCH_3_ and GO-PEG-NH_2_-treated cells, rather than the GO-PEG-COOH group.

To further quantitatively evaluate cell internalization of different PEG-GO nanosheets, flow cytometry was carried out to analyze the SSC value, which reflects the inner cell granularity and complexity [24]. Therefore, the internalization of nanomaterials will result in a rise of SSC value [28]. Our results showed that the treatment of GO-PEG-OCH_3_ and GO-PEG-NH_2_ samples significantly increased the SSC value in both hCorECs and hRCECs compared to the control (Figure 1E,F). Notably, the SSC values in the GO-PEG-OCH_3_ and GO-PEG-NH_2_ group were not significantly different, but higher than those of the GO-PEG-COOH group. This suggested that the cellular uptake efficacy of positively and neutrally charged PEG-GO nanosheets was higher than that of negatively charged PEG-GO nanosheets.

### 3.2. Cellular Biochemical Reactions

**Figure 2:**
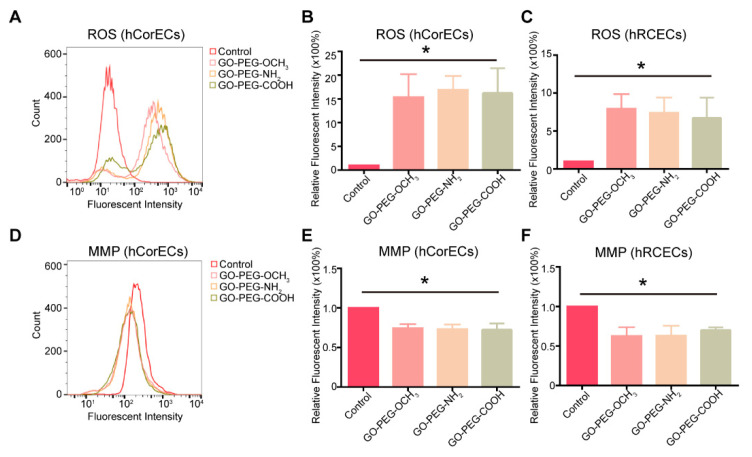
Cellular biochemical reactions of hCorECs and hRCECs exposed to PEG-GO nanosheets with different surface charges. (**A**) Fluorescence activated cell sorting (FACS) analysis of intracellular reactive oxygen species (ROS) in PEG-GO-treated hCorECs. (**B**,**C**) Group data of intracellular ROS (relative fluorescence intensity) of PEG-GO-treated hCorECs or hRCECs. (**D**) FACS analysis of mitochondrial membrane potential (MMP) in PEG-GO-treated hCorECs. (**E**,**F**) Group data of MMP (relative fluorescence intensity) of PEG-GO-treated hCorECs or hRCECs. The absence of PEG-GO samples served as the control. Bars show mean ± S.D. compared with control group: * *p* < 0.05.

In order to explore the effect of surface charges on cellular biochemical reactions, we firstly studied the oxidative stress reactions of hCorECs and hRCECs treated by PEG-GO nanosheets with different surface charges. After exposure to 50 μg/mL PEG-GO samples for 24 h, the intracellular ROS levels of hCorECs increased significantly compared with the control (Figure 2A). Quantitative analysis showed that the relative mean fluorescent intensity (MFI) of hCorECs treated by three kinds of PEG-GO nanosheets with different surface charges was not significantly different but was ~15 times higher than the MFI of the control group (Figure 2B). Consistently, MFI of hRCECs treated with PEG-GO samples was not significantly different but was ~8 times higher than that of the control (Figure 2C). These data suggested that the PEG-GO treatment induced oxidative stress, but independent on surface charge.

We then further studied the MMP of hCorECs and hRCECs after PEG-GO treatments. The results showed that the MMP of hCorECs and hRCECs was not significantly different among the three PEG-GO groups (Figure 2D–F). In addition, the MMP of PEG-GO-treated hCorECs decreased to ~70% compared with untreated cells, while it decreased to ~60% in hRCECs. These results suggested that the PEG-GO treatment caused mitochondrial dysfunctions, but independent on surface charge.

### 3.3. Gene Expression Profile Analysis

**Figure 3:**
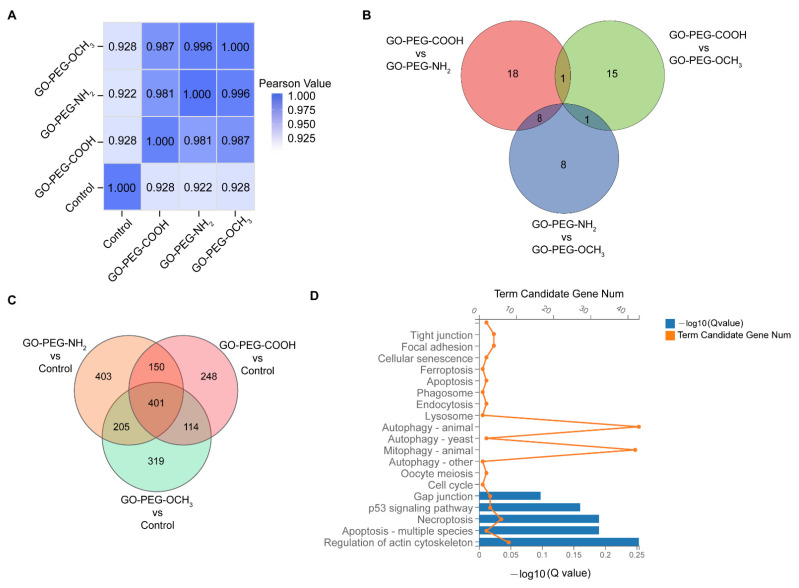
Gene expression profile analysis of hCorECs exposed to PEG-GO nanosheets. (**A**) Pearson correlation analysis of all gene expression in hCorECs of four groups. (GO-PEG-COOH vs. GO-PEG-NH_2_, 0.981; GO-PEG-OCH_3_ vs. GO-PEG-NH_2_, 0.996; GO-PEG-OCH_3_ vs. GO-PEG-COOH, 0.987; GO-PEG-COOH vs. Control, 0.928; GO-PEG-NH_2_ vs. Control, 0.922; GO-PEG-OCH_3_ vs. Control, 0.928). (**B**) Venn diagram showed the differentially expressed genes (DEGs) from paired comparisons among three PEG-GO groups. (**C**) Venn diagram showed the DEGs from paired comparisons between PEG-GO groups and control group. Genes with |log2 FC (fold change)| > 1 and adjusted *p* value ≤ 0.05 were considered to be DEGs. (**D**) The pathway analysis of 401 DEGs in terms of cellular processes based on Kyoto Encyclopedia of Genes and Genomes databases.

To further evaluate the effect of surface charge on gene expression, we used RNA-seq to study the global gene expression profiles of hCorECs treated by three kinds of PEG-GO samples (50 μg/mL) for 24 h. Untreated cells served as control. Using Pearson correlation analysis, we found that the correlation coefficients between three kinds of PEG-GO-treated cells were more than 0.98 (GO-PEG-COOH vs. GO-PEG-NH_2_, 0.981; GO-PEG-OCH_3_ vs. GO-PEG-NH_2_, 0.996; GO-PEG-OCH_3_ vs. GO-PEG-COOH, 0.987) (Figure 3A). There were no more than 30 DEGs identified via paired comparisons between PEG-GO-treated groups (GO-PEG-COOH vs. GO-PEG-NH_2_, 27; GO-PEG-OCH_3_ vs. GO-PEG-NH_2_, 17; GO-PEG-OCH_3_ vs. GO-PEG-COOH, 17; Figure 3B).

Compared with the control, we identified 1159, 913 and 1039 DEGs in GO-PEG-NH_2_, GO-PEG-COOH and GO-PEG-OCH_3_ groups, respectively (Figure 3C). Notably, the three PEG-GO groups shared 401 DEGs (Figure 3C). By KEGG database-based pathway analysis, the DEGs were enriched in pathways involving cellular processes, environmental information processing, genetic information processing, human diseases, metabolism and organismal systems fields. In terms of cellular process, the top 20 pathways were related to cell death and proliferation, endocytosis, autophagy and mitophagy (Figure 3D).

### 3.4. Ocular Surface and Intraocular Irritation Test

**Figure 4:**
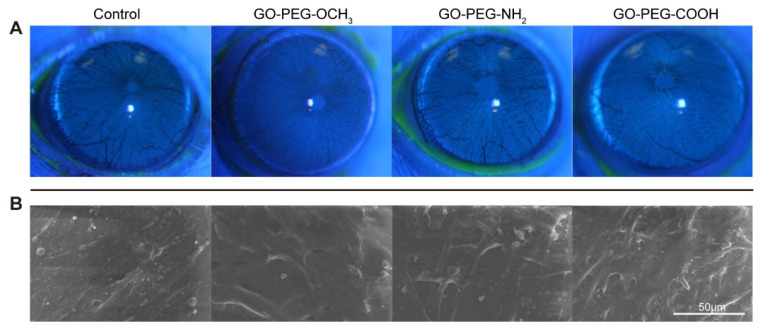
Ocular surface irritation test of PEG-GO nanosheets. (**A**) Corneal fluorescein staining of Sprague Dawley (SD) rats at 24 h post PEG-GO treatment. (**B**) Scanning electron microscope micrographs of the cornea at 24 h after PEG-GO treatment.

Considering the potential application of GO derivatives in eye diseases, we further studied the in vivo biocompatibility of the three PEG-GO samples with the ocular surface and inner eye. Firstly, 50 μg/mL PEG-GO samples were topically administered to ocular surface of SD rats. No signs of corneal opacity, conjunctival redness, abnormality of the iris or chemosis were observed. Corneal fluorescein-staining assay was performed to assess the toxicity to corneal epithelium, with positive fluorescein indicating the compromised epithelium. No visible positive fluorescein staining was found in any of the three PEG-GO groups (Figure 4A). Furthermore, the SEM assay showed that PEG-GO nanosheet treatment did not disturb the corneal ultrastructure (Figure 4B). These results indicated that none of the differently charged PEG-GO nanosheets induced significant irritation to ocular surface.

**Figure 5:**
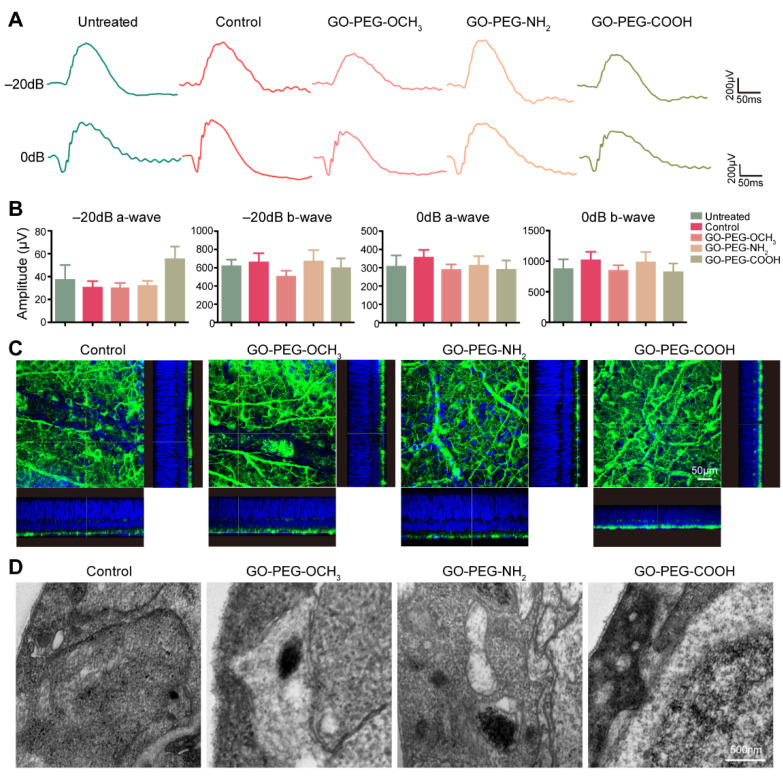
Intraocular irritation test of PEG-GO nanosheets. (**A**) Representative images of scotopic electroretinogram at flash intensity of −20 dB and 0 dB 4 weeks after intravitreal injection of PEG-GO nanosheets. (**B**) Group data of the amplitude of a-wave and b-wave. Bars show mean ± S.D. (**C**) Representative immunofluorescence images of the retinal whole mount with z scanning, showing the retinal ganglion cells (green). The cell nuclei were displayed in blue. Scale bars = 50 μm. (**D**) Representative ultrastructural images showing the retinal ganglion cells layer. Scale bars = 500 nm.

To further evaluate the intraocular toxicity, PEG-GO samples were intravitreally injected in LE rats, while the deionized-water-treated rats and untreated rats served as study. An ERG assay was conducted to analyze retinal functions 4 weeks after injection. The results showed that, compared to the control, both a- and b-wave amplitudes were not significantly changed in any of the three PEG-GO-treated groups under −20 dB and 0 dB stimulus (Figure 5A,B). Since the ganglion cell layer (GCL) was closest to the vitreous cavity, intravitreally injected PEG-GO samples would most likely interact with retinal ganglion cells (RGCs) in GCL. We therefore evaluated the morphology changes of RGCs via the retinal whole mount fluorescent staining of Tuj1, which is the marker of RGCs. Our results showed that there was no obviously different expression of Tuj1 detected in any PEG-GO-treated groups compared to control (Figure 5C). Consistently, TEM assays did not show any obvious changes in retinal structures among the four groups (Figure 5D). These data suggested that intravitreally injected PEG-GO samples with different surface charges did not induce any significant retinal function and structure impairment.

## 4. Discussion

The surface charge plays an important role in the nano–bio interactions. In this work, we studied the in vitro and in vivo ocular compatibility of positively, negatively and neutrally surface-charged PEGylated GO derivatives.

We firstly found that the cellular uptake efficacy of positively charged PEG-GO nanosheets was higher than that of the negatively charged PEG-GO nanosheets. Previous studies have also consistently demonstrated that the cellular uptake efficacy of positively charged nanomaterials, including GO, was higher than that of the negatively charged ones [7,14,29,30,31,32,33,34,35]. This could be attributed to the electrostatic interactions between the charged nanomaterials and the negatively charged cell membranes [36,37]. Positively charged nanomaterials might show stronger affinity to the negatively charged cell membranes and thus enhance the nano–bio interactions, while negatively charged ones are more likely to be suspended in the solvent and thus attenuate the interactions [36]. Notably, we also found that there was no obvious difference between positively and neutrally charged PEG-GO nanosheets in terms of cellular uptake. However, Tu et al. suggested that the positively charged reduced GO sheets showed a much higher cellular uptake efficiency than neutrally charged ones [7]. The difference might be that all GO nanosheets in our work were PEGylated. Previous studies reported that PEGylation could alter the distribution of nanoparticles and inhibit the interactions between nanomaterials and cells or proteins [9,36,37,38]. We inferred that PEGylation neutralized the electrostatic interaction and lowered the affinity between positively charged PEG-GO and negatively charged cell membranes.

Previous studies (including our own) have shown that GO and its derivatives induce nanotoxicity mainly via oxidative stress and mitochondrial dysfunctions [22,23,24,26,36]. In our study, we showed that the three kinds of PEG-GO nanosheets caused surface-charge-independent oxidative stress and mitochondrial dysfunctions. Next, we evaluated the role of surface charge on gene transcriptome. We found that the similarity between positively charged (GO-PEG-NH_2_) and neutrally charged (GO-PEG-OCH_3_) groups was highest among all three paired comparisons by Pearson correlation analysis. This was consistent with our previous results that positively charged PEG-GO nanosheets and neutrally charged PEG-GO nanosheets induced similar cytotoxicity in terms of cell viability [22], cellular uptake, oxidative stress and mitochondrial functions. In addition, previous studies have demonstrated that cellular uptake of nanomaterials, including GO derivatives, mainly occurred through phagocytosis and endocytosis pathways [7,16,34]. We also found consistently that the phagosome and endocytosis pathways were significantly changed in all the PEG-GO sample treated groups.

Although our previous and present in vitro studies both proved that PEG-GO nanosheets (50 μg/mL) were toxic to hCorECs and hRCECs [22], the in vivo studies showed that they had no notable toxicity on both ocular surface and inner eyes. This might be due to the differences of the uncontrolled factors, including actual exposure time and concentrations between in vitro and in vivo experiments. In addition, in the complex in vivo environment, the protective and restorative cells (such as immune cells) might protect the eyes against external irritation and mitigate or repair potential injury [39,40]. Previous studies have also consistently demonstrated that GO-based nanomaterials have good in vivo biocompatibility with ocular surface and inner eyes [17,18,19,20,21].

Therefore, according to the results above, we believe that surface charges of PEG-GO exerted negligible effect on its ocular compatibility, except for the cellular uptake. Considering the potential application of GO-based nanomaterials in drug delivery for ocular surface and intraocular diseases, our work will provide a basis for designing GO-based nanomaterials with high drug delivery efficiency and good biocompatibility.

## Figures and Tables

**Table 1:** Zeta potential (*ξ*) of water-dispersed PEG-GO in water (pH = 7.00) and DMEM, and hydrodynamic diameter (*D_h_*) for PEG-GO samples (mean ± S.D.).

		GO-PEG-OCH_3_	GO-PEG-COOH	GO-PEG-NH_2_
*ξ* (mV)	In water	−0.03 ± 0.01	−23.56 ± 1.26	+7.81 ± 0.61
	In DMEM	−0.26 ± 0.04	−27.56 ± 0.81	+6.79 ± 0.27
*D_h_* (nm)	In water	65.33 ± 3.06	64.67 ± 5.86	57.33 ± 5.69
	In DMEM	65.67 ± 4.16	66.67 ± 5.03	60.33 ± 4.04

Measurements above were performed with 50 μg/mL GO-PEG-OCH_3_, GO-PEG-COOH and GO-PEG-NH_2_ in water or DMEM, as described from our previous study [22]. Previously, we found that PEG-GO nanosheets with different surface charges exert similar toxicity, and the viability of hCorECs and hRCECs significantly decreased when the concentration of PEG-GO increased to 50 μg/mL and above [22]. In this study, we therefore comprehensively analyzed the cellular response of hCorECs and hRCECs exposure to 50 μg/mL PEG-GO samples, so that a large enough proportion of cells remained viable after 24 h of treatment in order to enable quantification [22].

## Data Availability

The data are available upon reasonable request from the corresponding authors.

## Supplementary Materials

The following supporting information can be downloaded at: https://www.mdpi.com/article/10.3390/nano12050735/s1, Figure S1: Atomic force microscopy (AFM) images of GO-PEG-OCH_3_, GO-PEG-COOH and GO-PEG-NH_2_, respectively. (The protocol of AFM measurement was performed as previously [22]).
