# Supplementary material for: Effects of the Surface Charge of Graphene Oxide Derivatives on Ocular Compatibility
Source: Nanomaterials (Basel). 2022 Feb 22;12(5):735. doi: 10.3390/nano12050735 (PMC8911648; doi:10.3390/nano12050735)
Supplement: Supplementary file 1 [file nanomaterials-12-00735-s001.zip › nanomaterials-1539787-supplementary.pdf]

Supplementary Materials

Effects of the Surface Charge of Graphene Oxide Derivatives on Ocular Compatibility

Liyuan Rong [1,2,†], Yan Fu [3,†], Qiyou Li [2], Xinji Yang [1], Yueyue Li [1], Liang Yan [4,*], Liqiang Wang [1,*] and Wei Wu [1,2,*]

1 Senior Department of Ophthalmology, the Third Medical Center of PLA General Hospital, Beijing 100143, China; liyuan_rong@outlook.com (L.R.); yangxinji68@sina.com (X.Y.); liyueyue_7909@163.com(Y.L.)
2 Department of Ophthalmology, Southwest Hospital, Third Military Medical University (Army Medical University), Chongqing 400038, China; lqy111lcy@126.com
3 The General Hospital of Western Theater Command, Chengdu 610000, China; fuyansmile@163.com
4 CAS Key Laboratory for Biomedical Effects of Nanomaterials and Nanosafety, Institute of High Energy Physics and National Center for Nanoscience and Technology, Chinese Academy of Sciences, Beijing 100049, China
* Correspondence: yanliang@ihep.ac.cn (L.Y.); liqiangw301@gmail.com (L.W.); wuwei1@301hospital.com.cn (W.W.)
† These authors contributed equally to this work.

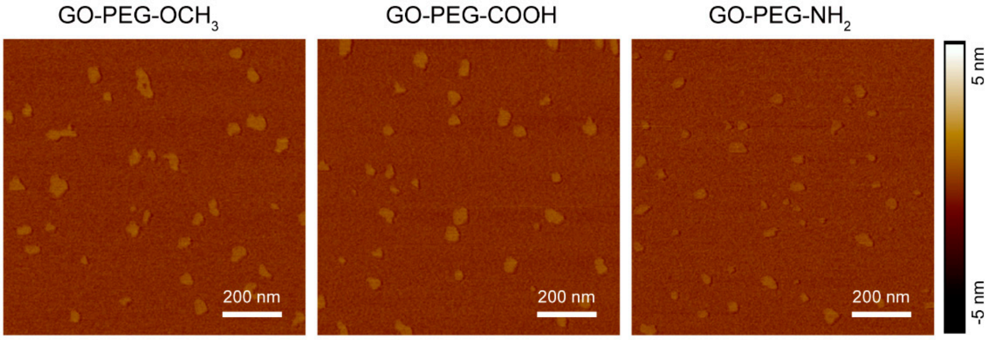

Figure S1. Atomic force microscopy (AFM) images of GO-PEG-OCH_3, GO-PEG-COOH and GO-PEG-NH_2, respectively. (AFM was performed as previously [22])
